# Genetic structure and demographic history of *Colletotrichum gloeosporioides sensu lato* and *C. truncatum* isolates from Trinidad and Mexico

**DOI:** 10.1186/1471-2148-13-130

**Published:** 2013-06-22

**Authors:** Sephra N Rampersad, Daisy Perez-Brito, Claudia Torres-Calzada, Raul Tapia-Tussell, Christine VF Carrington

**Affiliations:** 1 Department of Life Sciences, Faculty of Science and Technology, The University of the West Indies, St. Augustine. Trinidad and Tobago, West Indies; 2 Laboratorio GeMBio, Centro de Investigacion Científica de Yucatan A.C., Yucatan, Mexico; 3 Department of Pre-clinical Science, Faculty of Medical Sciences, The University of the West Indies, St. Augustine. Trinidad and Tobago, West Indies

**Keywords:** *Colletotrichum* spp., Genetic differentiation, Migration, Population structure

## Abstract

**Background:**

*C. gloeosporioides sensu lato* is one of the most economically important post-harvest diseases affecting papaya production worldwide. There is currently no information concerning the genetic structure or demographic history of this pathogen in any of the affected countries. Knowledge of molecular demographic parameters for different populations will improve our understanding of the biogeographic history as well as the evolutionary and adaptive potential of these pathogens. In this study, sequence data for ACT, GPDH, β-TUB and ITS gene regions were analyzed for *C. gloeosporioides sensu lato* and *C. truncatum* isolates infecting papaya in Trinidad and Mexico in order to determine the genetic structure and demographic history of these populations.

**Results:**

The data indicated that Mexico is the ancestral *C. gloeosporioides sensu lato* population with asymmetrical migration to Trinidad. Mexico also had the larger effective population size but, both Mexico and Trinidad populations exhibited population expansion. Mexico also had greater nucleotide diversity and high levels of diversity for each gene. There was significant sub-division of the Trinidad and Mexico populations and low levels of genetic divergence among populations for three of the four gene regions; β-TUB was shown to be under positive selection. There were also dissimilar haplotype characteristics for both populations. Mutation may play a role in shaping the population structure of *C. gloeosporioides sensu lato* isolates from Trinidad and from Mexico, especially with respect to the ACT and GPDH gene regions. There was no evidence of gene flow between the *C. truncatum* populations and it is possible that the Mexico and Trinidad populations emerged independently of each other.

**Conclusions:**

The study revealed relevant information based on the genetic structure as well as the demographic history of two fungal pathogens infecting papaya, *C. gloeosporioides sensu lato* and *C. truncatum*, in Trinidad and Mexico. Understanding the genetic structure of pathogen populations will assist in determining the evolutionary potential of the pathogen and in identifying which evolutionary forces may have the greatest impact on durability of resistance. Intervention strategies that target these evolutionary forces would prove to be the most practical.

## Background

*Colletotrichum gloeosporioides* is one of the most common and widely distributed plant pathogens in the world [1,2] and has been associated with a minimum of 470 different host genera, either as a pathogen or as an endophyte [3]. The fungus can cause quiescent infections that result in severe losses due to pre- and post-harvest disease in a wide range of fruit crops [4]. *C. gloeosporioides* is considered to be a species complex but for practical reasons, it may be simpler to refer to the taxa as a species complex rather than using intra-specific designations [5,6].

Anthracnose disease is one of the most economically important post-harvest diseases affecting papaya (*Carica papaya* L.) production worldwide [7]. *C. gloeosporioides sensu lato* is the pathogen most commonly associated with anthracnose of papaya. *C. acutatum* from papaya in Australia was identified as an ex-epitype strain by Simmonds in 1965, but it has not been reported in papaya since that time. There have been reports of *C. truncatum* (syn. *C. capsici*[6]) infecting papaya in Trinidad [8], Japan [9], Florida [10], Thailand [11] and in Mexico [12], but, anthracnose infection in papaya by *C. truncatum* is more widespread and severe in Mexico than in other affected countries.

**Figure 1:**
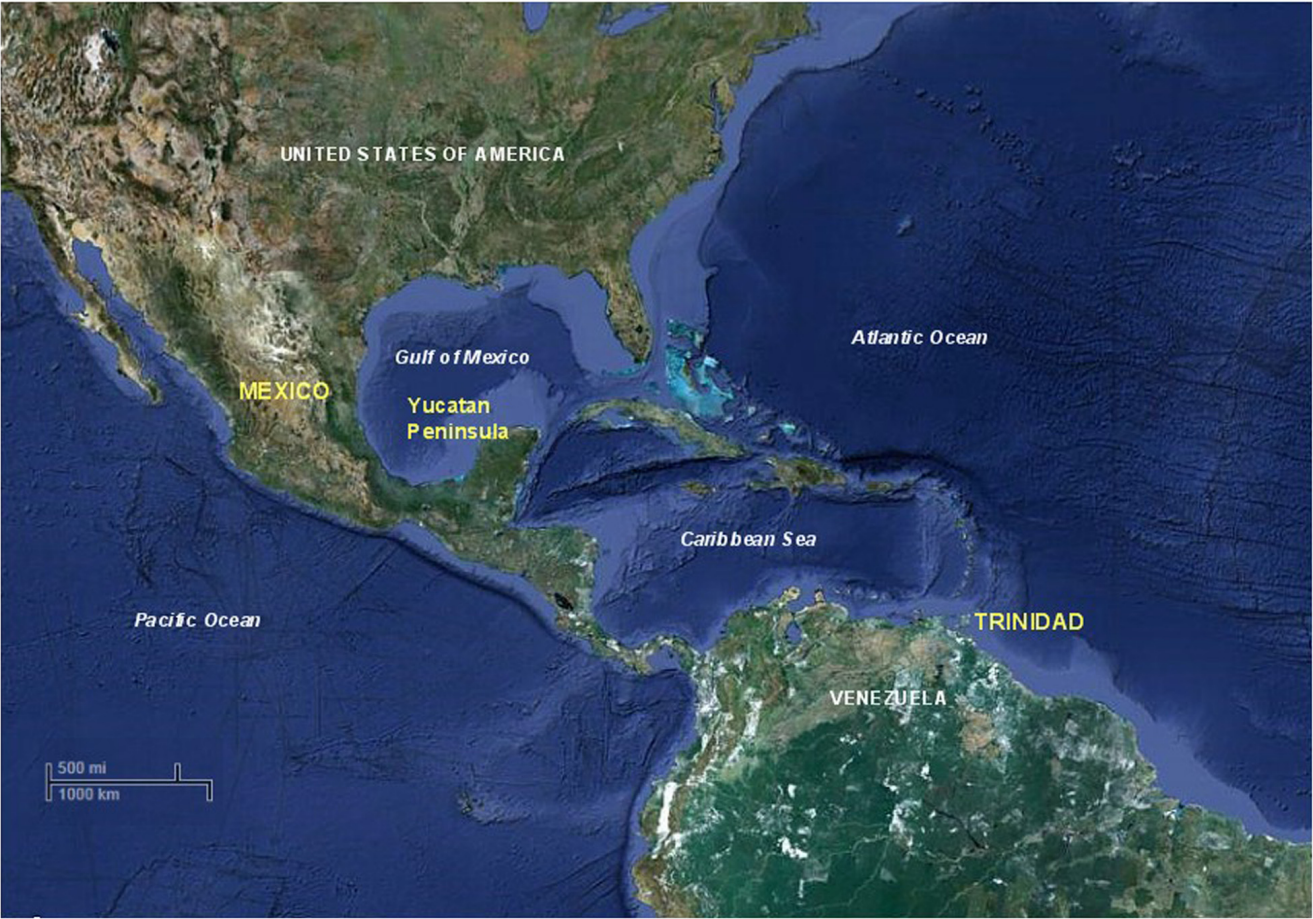
**Map of the Caribbean basin showing the locations of Trinidad and the Yucatan Peninsula, Mexico (Google Earth,**http://www.google.com/earth/index.html**).**

Global trade in papaya is dominated by USA, Canada and the EU [13]. Independently, production and export of papaya as fresh fruit in the Caribbean have increased over the last decade but the leading producers are Latin and Central America (50%), Asia and the Pacific (30%) and Africa (20%) [13]. Trinidad (10.5526°N, 61.3152°W) is one of the larger member islands of the Lesser Antilles in the West Indies. It lies in the Caribbean Sea, off the north-eastern coast of Venezuela; east of Trinidad is the Atlantic Ocean (Figure 1). It lies approximately 2893 km from Mexico. Papaya is an important fruit crop in Trinidad and two cultivars are commonly grown, ‘Red lady’ and ‘Tainung No. 2 - F1 hybrid’. Mexico is ranked among the leading global producers of papaya [13,14]; Chiapas, Veracruz, Oaxaca and the Yucatan Peninsula are the main producing states [15]. ‘Maradol’ is the most widely grown cultivar and the fruit is exported to several countries but mainly to USA and Canada. The Yucatan Peninsula (20.8333°N, 89.0000°W) in southern Mexico separates the Gulf of Mexico from the Caribbean Sea. It lies in south-eastern Mexico and is north of Guatemala and Belize. Off of the eastern coast is the Caribbean Sea and to the west is the Pacific Ocean.

It is widely believed that papaya originated from the Caribbean coast of Central America [7,16] through natural hybridization between *Carica peltate* and another wild species [17]. Historical records indicate that papaya seeds were taken to the Panama coast and to the Dominican Republic before 1525 and cultivation spread throughout the other islands of the Caribbean by 1616 [7]. There is currently no information regarding the population structure or demographic history of *C. gloeosporioides sensu lato* and *C. truncatum* as pathogens of *C. papaya* in any of the affected countries. Cannon et al. [5] purported that unique *C. gloeosporioides sensu lato* genotypes may become adapted to occupy specific geographical areas and which may be associated with host co-evolution. The genetic diversity of *C. gloeosporioides sensu lato* isolates is large where native or naturalized host species occur compared to locations where the host species has been recently introduced [18]. One of the underlying hypotheses of this study is the possible inter-dependence of the phylogeographic histories of *C. gloeosporioides sensu lato*, *C. truncatum* and the host; this hypothesis is, in part, based on the assumption that the Caribbean coast of Mexico is the centre of origin of the host species and the first distribution of the host species occurred in the Caribbean by the 16^th^ century.

One approach for deriving meaningful information on population genetics is by comparing multiple nucleotide sequences, including arbitrary regions [19], and subsequent assessment of calculated patterns of diversity, tests of selection (Tajima’s *D*, Fu and Li’s F* and D* statistics), gene flow, linkage disequilibrium and migration [20]. Many potential markers are inappropriate for certain applications due to low sequence variability (e.g., *rpo*B, *ndh*J, *acc*D, *atp*B, COX1, 5.8S rRNA) [21]. A comparison of genic regions that exhibit the greatest level of polymorphisms to allow intra-specific genotyping is preferred [22,23]. Multi-gene phylogenetics have been used to systematically characterize *Colletotrichum* species relationships [24-28]. For example, Prihastuti et al. [28] used six genes, the nuclear rDNA internal transcribed spacer (ITS) region, partial actin (ACT), β-tubulin (β-TUB), calmodulin (CAL), glutamine synthetase (GS) and glyceraldehyde 3-phosphate dehydrogenase (GPDH) to study *C. gloeosporioides sensu lato* and concluded that species relationships could be resolved.

Knowledge of molecular demographic parameters, such as the effective population sizes, levels of species divergence, and rates of gene flow and migration patterns between populations will elucidate the biogeographic histories of the species, the evolutionary and adaptive potential of these pathogens; information on the genetic structure of these populations can also assist in the development of disease management strategies i.e. pesticide use, predicting resistance breakdown, production of disease resistant transgenics and in streamlining cultural practices [29-32]. Understanding the evolution of epidemics ensures that the full spectrum of genetic variability of the pathogen population is captured or represented when screening against genetic host tolerance, fungicide sensitivity, or any research application where pathogen diversity is relevant to the outcome of any proposed intervention strategy.

The objectives of this study were to (i) compare the genetic and population structure of isolates of *C. gloeosporioides sensu lato* and *C. truncatum* from Mexico and Trinidad and (ii) determine the demographic history of these isolates based on multi-locus sequence data.

## Results

Isolate collection data for Trinidad and Mexico is summarized in Table 1.

**Table 1:** Summary of isolate collection data for Trinidad and Mexico

**Isolate**	**County/State**	**Location**	**Morphotype**	**Cultivar**	**Year**
PAW-Cg-18.1-T	Victoria	South Trinidad	M3 (T&T)	Tainung No. 2-F1 hybrid	2011
PAW-Cg-18.2-T	Victoria	South Trinidad	M3 (T&T)	Tainung No. 2-F1 hybrid	2011
PAW-Cg-9.1-T	Victoria	South Trinidad	M3 (T&T)	Tainung No. 2-F1 hybrid	2011
PAW-Cg-4.2-T	Victoria	South Trinidad	M3 (T&T)	Tainung No. 2-F1 hybrid	2011
PAW-Cg-5.1-T	Victoria	South Trinidad	M3 (T&T)	Tainung No. 2-F1 hybrid	2011
PAW-Cg-11.2-T	Victoria	South Trinidad	M3 (T&T)	Tainung No. 2-F1 hybrid	2011
PAW-Cg-8.1-T	Victoria	South Trinidad	M3 (T&T)	Red Lady	2011
PAW-Cg-10.1-T	Victoria	South Trinidad	M3 (T&T)	Red Lady	2011
PAW-Cg-6.1-T	Victoria	South Trinidad	M3 (T&T)	Tainung No. 2-F1 hybrid	2011
PAW-Cg-20.1-T	Victoria	South Trinidad	M3 (T&T)	Tainung No. 2-F1 hybrid	2011
PAW-Cg-20.2-T	Victoria	South Trinidad	M3 (T&T)	Tainung No. 2-F1 hybrid	2011
PAW-Cg-19.2-T	Victoria	South Trinidad	M3 (T&T)	Tainung No. 2-F1 hybrid	2011
PAW-Cg-15.2-T	Victoria	South Trinidad	M3 (T&T)	Tainung No. 2-F1 hybrid	2011
PAW-Cg-14.1-T	Victoria	South Trinidad	M3 (T&T)	Tainung No. 2-F1 hybrid	2011
PAW-Cg-8.11-T	Victoria	South Trinidad	M3 (T&T)	Red Lady	2011
PAW-Cg-21.1-T	Mayaro	South Trinidad	M4 (T&T)	Red Lady	2011
PAW-Cg-11.1-T	Mayaro	South Trinidad	M2 (T&T)	Tainung No. 2-F1 hybrid	2011
PAW-Cg-4.1-T	Mayaro	South Trinidad	M2 (T&T)	Tainung No. 2-F1 hybrid	2011
PAW-Cg-16.1-T	Mayaro	South Trinidad	M2 (T&T)	Tainung No. 2-F1 hybrid	2011
PAW-Cg-11.11-T	Mayaro	South Trinidad	M2 (T&T)	Red Lady	2011
PAW-Cg-100-T	St. George West	North Trinidad	M1 (T&T)	Red Lady	2011
PAW-Cg-101-T	St. George West	North Trinidad	M3 (T&T)	Red Lady	2011
PAW-Cg-102-T	St. George West	North Trinidad	M3 (T&T)	Tainung No. 2-F1 hybrid	2011
PAW-Cg-103-T	St. George West	North Trinidad	M3 (T&T)	Tainung No. 2-F1 hybrid	2011
PAW-Cg-104-T	St. George West	North Trinidad	M2 (T&T)	Tainung No. 2-F1 hybrid	2011
PAW-Cg-105-T	St. George West	North Trinidad	M2 (T&T)	Red Lady	2011
PAW-Cg-106-T	St. George East	North Trinidad	M4 (T&T)	Red Lady	2011
PAW-Cg-107-T	St. George East	North Trinidad	M4 (T&T)	Red Lady	2011
PAW-Cg-108-T	St. George East	North Trinidad	M3 (T&T)	Tainung No. 2-F1 hybrid	2011
PAW-Cg-109-T	St. George East	North Trinidad	M3 (T&T)	Tainung No. 2-F1 hybrid	2011
CGP1	Quintana Roo	South Mexico	M4 (Mx)	Maradol	2010
CGP2	Quintana Roo	South Mexico	M5 (Mx)	Maradol	2010
CGP3	Quintana Roo	South Mexico	M5 (Mx)	Maradol	2011
CGP4	Quintana Roo	South Mexico	M6 (Mx)	Maradol	2011
CGP5	Quintana Roo	South Mexico	M7 (Mx)	Maradol	2011
CGP6	Quintana Roo	South Mexico	M7 (Mx)	Maradol	2011
CGP7	Quintana Roo	South Mexico	M6 (Mx)	Maradol	2011
CGP8	Quintana Roo	South Mexico	M5 (Mx)	Maradol	2011
CGP14	Yucatán	South Mexico	M8 (Mx)	Maradol	2011
CGP15	Yucatán	South Mexico	M6 (Mx)	Maradol	2011
CGP16	Yucatán	South Mexico	M8 (Mx)	Maradol	2011
CGP17	Yucatán	South Mexico	M8 (Mx)	Maradol	2011
CGP18	Yucatán	South Mexico	M8 (Mx)	Maradol	2011
CGP19	Quintana Roo	South Mexico	M8 (Mx)	Maradol	2011
CGP20	Quintana Roo	South Mexico	M6 (Mx)	Maradol	2011
CGP21	Quintana Roo	South Mexico	M8 (Mx)	Maradol	2011
CGP22	Quintana Roo	South Mexico	M4 (Mx)	Maradol	2011
PAW-Ct-1-T	St. George West	North Trinidad	M5 (T&T)	Red Lady	2011
PAW-Ct-2-T	St. George West	North Trinidad	M5 (T&T)	Tainung No. 2 F1 hybrid	2011
PAW-Ct-3-T	St. George West	North Trinidad	M5 (T&T)	Tainung No. 2 F1 hybrid	2011
PAW-Ct-4-T	St. George West	North Trinidad	M5 (T&T)	Tainung No. 2 F1 hybrid	2011
PAW-Ct-5-T	St. George West	North Trinidad	M5 (T&T)	Tainung No. 2 F1 hybrid	2011
PAW-Ct-6-T	Victoria	South Trinidad	M5 (T&T)	Red Lady	2011
PAW-Ct-7-T	Victoria	South Trinidad	M5 (T&T)	Red Lady	2011
PAW-Ct-8-T	St. George West	North Trinidad	M5 (T&T)	Tainung No. 2 F1 hybrid	2011
CCP1	Campeche	South Mexico	M3 (Mx)	Maradol	2011
CCP4	Campeche	South Mexico	M1 (Mx)	Maradol	2011
CCP6	Quintana Roo	South Mexico	M3 (Mx)	Maradol	2011
CCP10	Quintana Roo	South Mexico	M3 (Mx)	Maradol	2011
CCP11	Quintana Roo	South Mexico	M3 (Mx)	Maradol	2011
CCP12	Quintana Roo	South Mexico	M3 (Mx)	Maradol	2011
CCP14	Quintana Roo	South Mexico	M3 (Mx)	Maradol	2011
CCP15	Quintana Roo	South Mexico	M3 (Mx)	Maradol	2011
CCP16	Quintana Roo	South Mexico	M3 (Mx)	Maradol	2011
CCP17	Quintana Roo	South Mexico	M3 (Mx)	Maradol	2011

Key for Morphotype Designation for Trinidad and Mexico Isolates:-

M1 (T&T): Olive-colored colony with dark grey-colored conidial mass in center.

M2 (T&T): Cream to light salmon-colored colony with no visible conidial mass in center.

M3 (T&T): Cream to pink-colored colony with greyish-colored conidial mass in center.

M4 (T&T): White flocculose colony with no visible conidial mass in center.

M5 (T&T): Tan-colored colony with no visible conidial mass.

M1 (Mx): Light salmon to pale grey colony, with conidial mass in center.

M2 (Mx): White to light salmon colony, with conidial masses produced in concentric rings.

M3 (Mx): Pale grey to black colony, with conidial masses produced in concentric rings.

M4 (Mx): White to orange colony, with orange conidial mass in center.

M5 (Mx): White flocculose colony, with orange conidial mass in center.

M6 (Mx): White to pale grey colony, with orange conidial mass produced in concentric rings.

M7 (Mx): White flocculose colony, with no visible conidial mass.

M8 (Mx): Olive to black colony, with no visible conidial mass.

### DNA divergence

Measures of DNA divergence enabled gene-by-gene comparisons of the level of genetic diversity in each population under study. *C. gloeosporioides sensu lato* isolates from Trinidad had more segregating sites (*S*) in the ACT and ITS sequences than those from Mexico as well as a greater number of mutations (Eta) and haplotypes (h) than Mexico. Haplotype diversity (Hd_T_) was highest for ACT but there were near equivalent values for the other three genes. The highest number of haplotypes (h_T_) was obtained for the ACT gene, followed by GPDH, β-TUB and ITS (Table 2(a)). Only one haplotype of the GPDH gene was shared between the two populations.

**Table 2:** **Summary of the number of isolates, length of gene fragment, haplotype number, haplotype diversity, and haplotype frequencies for Trinidad and Mexico populations of *Colletotrichum gloeosporioides sensu lato *and *C. truncatum***

	**ACT**		**GPDH**		**B-TUB**		**ITS**	
	**Trinidad**	**Mexico**	**Trinidad**	**Mexico**	**Trinidad**	**Mexico**	**Trinidad**	**Mexico**
(a)								
# isolates	30	17	30	17	30	17	30	17
Common sequence length, nt	174	174	221	221	301	301	496	496
No. of haplotypes, h	14	10	12	6	3	6	10	7
Haplotype diversity, *H*d	0.922	0.863	0.852	0.800	0.576	0.768	0.786	0.811
**Pairwise comparisons**								
Total # haplotypes, hT	23		16		9		14	
Total haplotype diversity, *H*dT	0.945		0.849		0.816		0.822	
(b)								
# isolates	8	13	8	13	8	13	8	13
Common sequence length, nt	174	174	221	221	301	301	496	496
No. of haplotypes, h	2	2	1	3	1	4	1	4
Haplotype diversity, *H*d	0.571	0.667	0	0.356	0	0.533	0	0.378
**Pairwise comparisons**								
Total # haplotypes	4		2		5		4	
Total haplotype diversity, *H*dt	0.676		0.209		0.679		0.634	

The *C. gloeosporioides sensu lato* isolates from Mexico had a higher number of nucleotide differences (k) and consequentially, higher nucleotide diversity (Pi) than Trinidad for all four genes. A comparison of DNA divergence parameters for the four genes indicated that GPDH had the highest cumulative number of polymorphic sites, total number of mutations and the highest average number of nucleotide differences followed by ACT, ITS and β-TUB had the lowest. There were no fixed differences among the four genes. Watterson’s theta (*θ*-W per site and per sequence) was also higher for ACT and ITS (Trinidad) while theta was higher for GPDH and β-TUB (Mexico). Generally, the conservation threshold (CT) was highest for β-TUB and ITS genes (Trinidad and Mexico) and lowest for GPDH (Mexico). There were no shared mutations in the β-TUB gene; GPDH and ITS had a near equivalent number of shared mutations and ACT had the least number_._ The average and net number of nucleotide substitutions per site between populations were highest for GPDH, followed by ACT, β-TUB and ITS (Da and Dxy(JC)). The average number of nucleotide differences (K_T_) and consequentially, nucleotide diversity (Pi_T_) were highest for GPDH, followed by ACT (Table 3(a)).

**Table 3:** **Summary of the DNA divergence values for Trinidad and Mexico populations of (a) *Colletotrichum gloeosporioides sensu lato *and (b) *C. truncatum***

	**ACT**		**GPDH**		**β-TUB**		**ITS**	
	**Trinidad**	**Mexico**	**Trinidad**	**Mexico**	**Trinidad**	**Mexico**	**Trinidad**	**Mexico**
(a)								
# polymorphic sites, S	54	42	35	94	5	38	58	25
Total # mutations, Eta	60	44	38	12	5	40	58	26
Theta (per site), S	0.109	0.085	0.062	0.288	0.004	0.039	0.033	0.015
Theta (per site), Eta	0.109	0.085	0.063	0.278	0.004	0.040	0.032	0.016
Theta (per site), Pi	0.044	0.079	0.036	0.206	0.009	0.039	0.012	0.015
Theta-W (per site)	0.087	0.074	0.054	0.178	0.004	0.037	0.031	0.015
Theta-W (per sequence)	13.631	11.839	8.912	26.496	1.273	10.711	14.769	7.047
Conservation threshold, CT	0.52	0.580	0.45	0.38	0.99	0.91	0.92	0.92
Ave. # nucleotide differences, k	6.49	11.168	4.778	24.111	2.557	10.063	5.414	7.132
Nucleotide diversity, Pi	0.042	0.072	0.035	0.162	0.009	0.037	0.011	0.015
Nucleotide diversity, Pi(JC)	0.045	0.081	0.036	0.221	0.009	0.039	0.012	0.016
**Pairwise comparisons**								
Ave # nucleotide differences, *K*T	8.833		13.432		6.541		6.366	
Total nucleotide diversity, PiT	0.057		0.091		0.023		0.013	
(b)								
# polymorphic sites, S	4	6	0	1	0	10	0	3
Total # mutations, Eta	4	6	0	1	0	10	0	3
Theta (per site), S	0.008	0.009	0.000	0.002	0.000	0.016	0.000	0.002
Theta (per site), Eta	0.008	0.009	0.000	0.002	0.000	0.016	0.000	0.002
Theta (per site), Pi	0.012	0.006	0.000	0.002	0.000	0.009	0.000	0.001
Theta-W (per site)	0.008	0.009	0.000	0.002	0.000	0.016	0.000	0.002
Theta-W (per sequence)	1.543	2.121	0.000	0.353	0.000	3.535	0.000	1.060
Conservation threshold, CT	0.710	0.770	0.670	0.670	0.960	0.970	0.930	0.930
Ave. # nucleotide differences, k	2.286	0.2	0.000	0.356	0.000	1.800	0.000	0.756
Nucleotide diversity, Pi	0.012	0.001	0.000	0.002	0.000	0.013	0.000	0.002
Nucleotide diversity, Pi(JC)	0.012	0.001	0.000	0.002	0.000	0.014	0.000	0.002
**Pairwise comparisons**								
Ave # nucleotide differences, *K*T	5.758		0.209		36.765		1.268	
Total nucleotide diversity, PiT	0.029		0.001		0.272		0.003	

*C. truncatum* isolates from Trinidad had no polymorphisms in the sequences of three gene regions (GPDH, β-TUB and ITS) analyzed and as such certain population genetics statistics could not be computed. The Mexico population had greater diversity for all parameters. None of the haplotypes were shared between the two populations except for one haplotype of the GPDH gene. The conservation threshold was highest for β-TUB and ITS and near equivalent for Trinidad and Mexico. GPDH had the lowest conservation threshold value for both populations (Tables 2(b) and 3(b)).

### Genetic differentiation and population structure

An assessment of the genetic differentiation was conducted to determine the level of sub-population structuring for all isolates and for each gene analyzed. Hudson’s *S*_nn_ statistic also suggested that the two populations were highly structured; *S*_nn_ values were significant and close to 1.0 for two genes (ACT and β-TUB). The *S*_nn_ value for the GPDH gene region was intermediate between 0.5 and 1.0 (Table 3(a)). For the ITS gene regions, however, the *S*_nn_ value was closer to 0.5 than to 1.0. Hudson’s *S*_nn_ statistic indicated that the *C. truncatum* populations were highly structured (*S*_nn_ values were close to 1.0) for ACT, β-TUB and ITS gene regions but, the *S*_nn_ value for the GPDH gene region was close to 0.5 (Table 3(b)).

**Figure 2:**
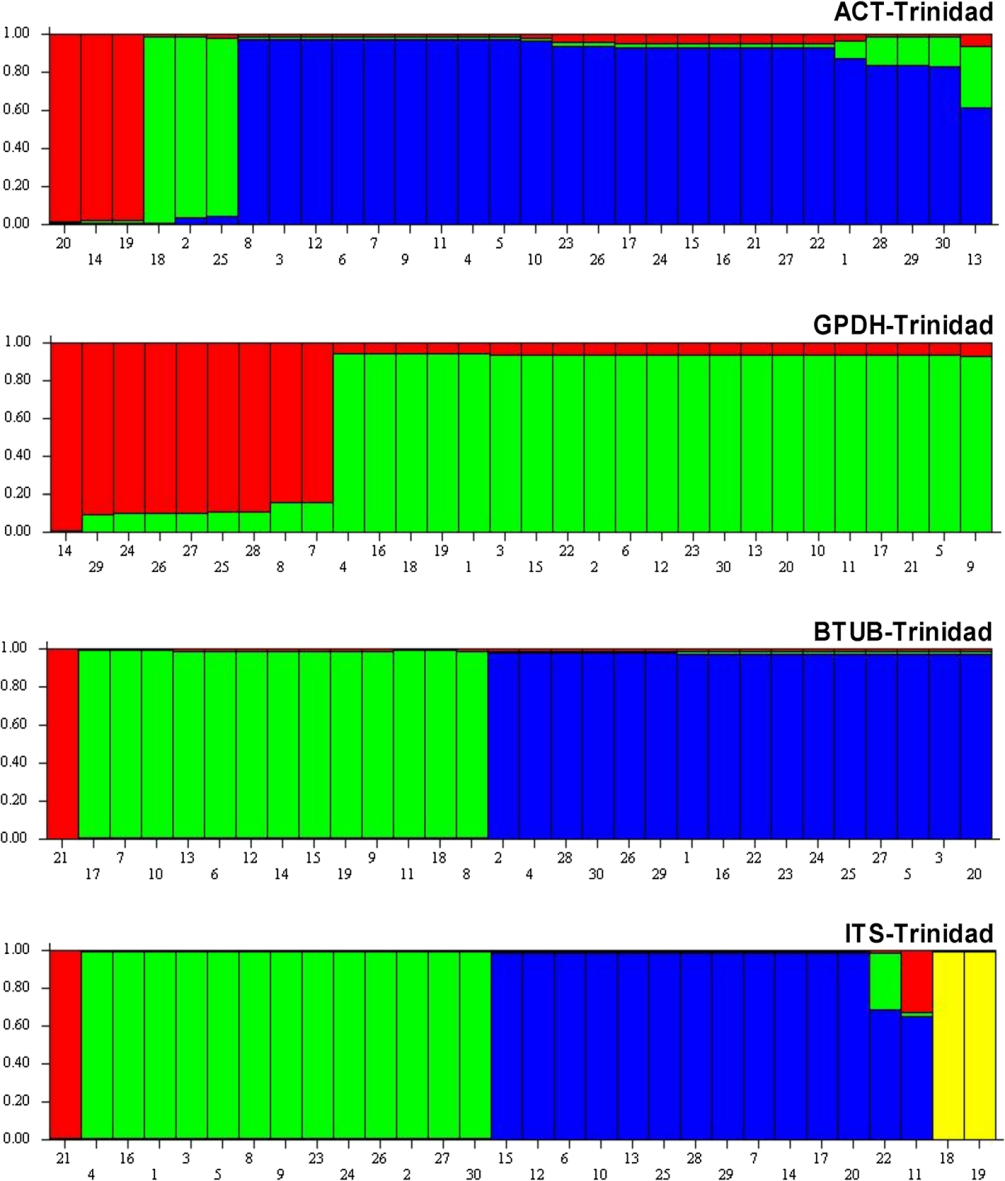
**Bar plot shows Q (estimated membership coefficients by posterior probability) for each gene region for the Trinidad population of *Colletotrichum gloeosporioides sensu lato.*** The height of each region within an individual bar is the measure of proportional affiliation. Individuals with membership coefficients of q_i_ ≥ 0.7 were assigned to a specific genetic cluster.

**Figure 3:**
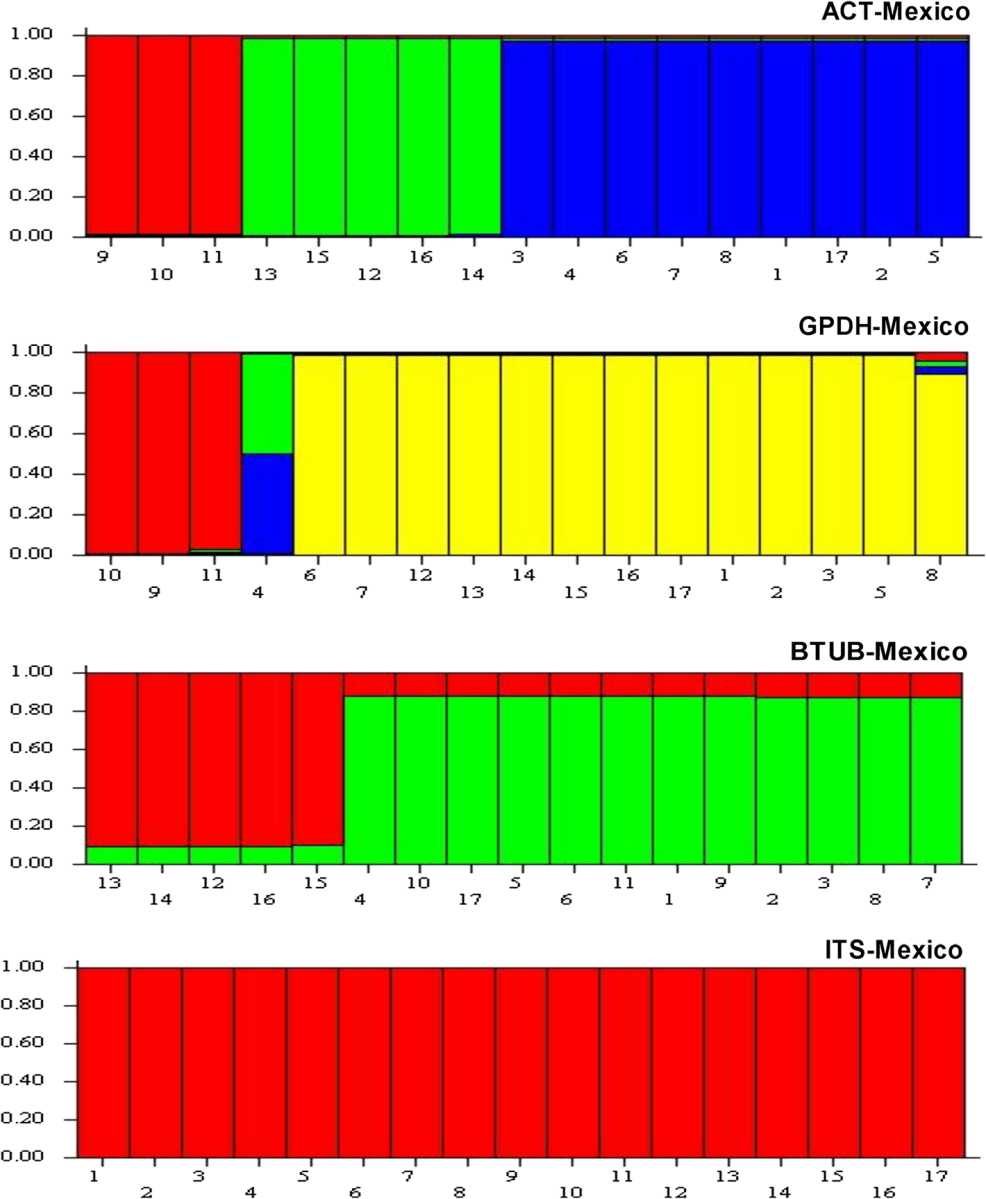
**Bar plot shows Q (estimated membership coefficients by posterior probability) for each gene region for the Mexico population of *Colletotrichum gloeosporioides sensu lato.*** The height of each region within an individual bar is the measure of proportional affiliation. Individuals with membership coefficients of q_i_ ≥ 0.7 were assigned to a specific genetic cluster.

STRUCTURE was used to determine population sub-division for all gene regions. Individuals with membership coefficients of q_i_ ≥ 0.7 were assigned to a specific genetic cluster. Sub-division was detected for all gene regions of all isolates of the *C. gloeosporioides sensu lato* Trinidad population with a maximum of three genetic clusters for ITS and two clusters for the other three gene regions. Sub-division was also detected in the *C. gloeosporioides sensu lato* Mexico population with a maximum of two genetic clusters for ACT, GPDH and β-TUB. However, sub-division was not detected for the ITS gene region for the Mexico isolates and a single population appeared to predominate (Figures 2 and 3). Evidence of gene flow was apparent for the two populations of *C. gloeosporioides sensu lato* isolates for three of the four genes (Table 4(a)).

**Table 4:** **Population structure test statistics *H***_**S**_**, *K***_S_*, Z* and *S*_**nn**_**, *N***_**m **_**and the value of Nei’s *G***_**ST **_**for comparisons between populations of (a) *Colletotrichum gloeosporioides sensu lato *and (b) *C. truncatum***

	**ACT**	**GPDH**	**β-TUB**	**ITS**
(a)				
***G***_**ST**_	0.05558	0.03291	0.21959	0.03294
***N***_**m**_	4.8	7.13	1.03	7.52
***H***_**S**_	0.89011****	0.81308**	0.61474****	0.77559**
***K***_S_*	1.47418**	1.63659**	0.98041****	1.03991**
Z*	5.84568**	5.93703**	5.74767****	5.92872**
***S***_**nn**_	0.93457 ****	0.73913****	1.00****	0.64739**
(b)				
***G***_**ST**_	0.45403	0.07235	0.55014	0.65626
***N***_**m**_	0.3	0.4	0.2	0.1
***H*s**	0.35918****	0..20317	0.30476****	0.21587****
*K*s*	0.47336****	0.14083	0.40415****	0.22520****
Z*	3.67783****	4.276	3.49141****	3.61505****
***S***_**nn**_	1.000 ****	0.52593	1.00****	0.97222****

* *P* ≤ 0.05.

** *P* ≤ 0.01.

*** *P* ≤ 0.001.

**** *P* ≤ 0.0001.

**Figure 4:**
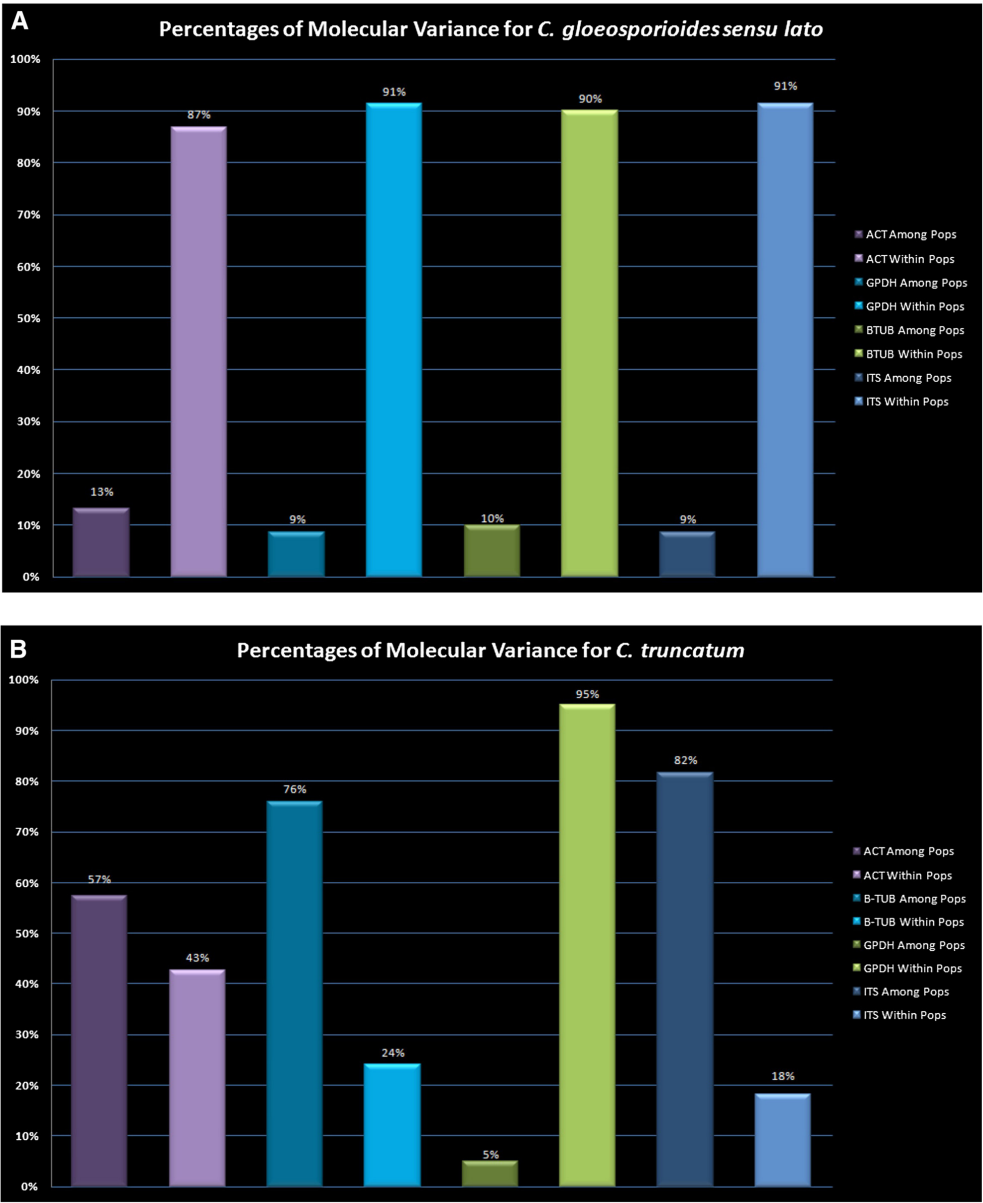
**Hierarchical analysis of molecular variation (AMOVA) as a measure of population differentiation for Trinidad and Mexico populations of A, *Colletotrichum gloeosporioides sensu lato *and B, *C. truncatum.*** Values are statistically significant at *P* ≤ 0.01 after 9,999 re-samplings.

With respect to *C. gloeosporioides sensu lato* isolates, AMOVA indicated population sub-structuring for the Trinidad and Mexico populations; of the total observed variation in the ACT gene, 13% was between populations and 87% was within populations. For the other three genes, there was 9 to 10% variation between populations and 90 to 91% variation within populations (Figure 4A).

For *C. truncatum* isolates, after 1,000 permutations, *G*_ST_ values were high for three genes (ACT, β-TUB and ITS) and ranged from 0.454 to 0.656 suggesting a high level of genetic differentiation between *C. truncatum* populations. However, AMOVA identified that of the total observed variation in the ACT gene, 57% was between populations and 43% was within populations; in the GPDH gene it was 5% and 95%; in the β-TUB gene it was 71% and 24%; in the ITS gene it was 82% and 18%, respectively (Figure 4B). There was no gene flow between the two *C. truncatum* populations for any of the genes (Table 4(b)).

There was no significant linear relationship between genetic and geographic distances and therefore, a poor fit of the isolation by distance model for *C. gloeosporioides sensu lato* isolates from Trinidad and Mexico. Isolation by distance could not explain the genetic structure of the Trinidad and Mexico populations (Table 5).

**Table 5:** **Mantel test of isolation by distance (correlation of genetic distance with geographic distance) for Trinidad and Mexico populations of *Colletotrichum gloeosporioides sensu lato***

	**Regression equation**	**R**^**2**^**value**	***P *- value**
**ACT**	“-0.3344x + 9.4049”	0.0227	0.324
**GPDH**	“-0.001x + 2.09”	0.2435	0.315
**β-TUB**	“-0.001x + 2.09”	0.2435	0.315
**ITS**	“-0.0001x + 1.1108”	0.0046	0.505

Significance level at *P* ≤ 0.05.

### Linkage disequilibrium

Significant LD was found for all four genes in *C. gloeosporioides sensu lato* populations from Trinidad and Mexico. There was a higher percentage of significant pairwise comparisons for three genes (ACT, β-TUB, ITS) for the Trinidad population than for the Mexico population. For Mexico, the highest percentage of LD was found in the GPDH gene (54.29%). For the Trinidad population, the highest percentage of LD was found in the β-TUB gene (100.00%) (Table 6). LD could not be computed for *C. truncatum* isolates.

**Table 6:** **Linkage disequilibrium (LD) tests for Trinidad and Mexico populations of *Colletotrichum gloeosporioides sensu lato***

		**# pairwise comparisons**	**# significant pairwise comparisons**	**% loci under LD**
**ACT**	Mexico	28	6	21.43
	Trinidad	91	29	31.87
**GPDH**	Mexico	105	57	54.29
	Trinidad	91	26	28.57
**β-TUB**	Mexico	28	6	21.43
	Trinidad	10	10	100.00
**ITS**	Mexico	6	1	16.70
	Trinidad	21	9	42.86

### Population size changes

(a) Tests of neutrality-Tajima’s *D* and Fu and Li’s F* and D* tests

Neutrality tests were used as an indication of recent population expansion. Strongly negative and significant values suggest recent population expansion or selection. Tajima’s *D* values were negative for all four genes in the *C. gloeosporioides sensu lato* Mexico population and for three genes (ACT, GPDH and ITS) in the *C. gloeosporioides sensu lato* Trinidad population. Only β-TUB (Trinidad) had positive *D*-values but these were not significant at *P* ≤ 0.05. For both Trinidad and Mexico populations, three genes, ACT, GPDH and ITS, had Tajima’s *D*-values that were less than zero and were significantly different from the neutral assumption which suggest recent population expansion or selection. The F* statistic was negative and significantly (*P* ≤ 0.02) deviated from the assumption of neutrality for three genes (ACT, β-TUB and ITS) in the Trinidad population. The D* statistic significantly deviated from the null hypothesis for ITS (Trinidad) at *P* ≤ 0.02, but ACT (Trinidad) and GPDH (Mexico) deviated significantly at *P* ≤ 0.05 and were large negative values (Table 7(a)).

The *C. truncatum* population from Trinidad could not be assessed because there were no polymorphisms for three of the four genic regions. Data for only the ACT gene (Trinidad) was analyzed, but none of the tests of neutrality were significant at the stipulated alpha levels. Tajima’s D and Fu and Li’s F* and D* statistics were negative and significant at *P* ≤ 0.05 and *P* ≤ 0.02 levels respectively for the β-TUB (Mexico) (Table 7(b)).

(b) Mismatch distribution and demographic expansion

Mismatch distribution was used to determine patterns of demographic expansion for all populations and to test the null hypothesis of population growth. With respect to *C. gloeosporioides sensu lato* isolates, none of the sums of squared deviations (SSD) of mismatch distribution was significant for the ACT and GPDH gene regions indicating a fit to the demographic expansion model tested. These results suggest that the deviation from neutrality was due to recent population expansion. For both populations, the sums of squared deviations (SSD) of mismatch distribution for β-TUB and ITS gene regions were significant which provides evidence to reject the null hypothesis of recent population expansion (Table 7(a)).

Based on the demographic data for β-TUB, the potential role for selection in shaping substitution rates within the β-TUB -protein coding gene was assessed using DNASP by comparing the number of synonymous and non-synonymous substitutions within populations to those between populations. The Ka to Ks ratio was greater than one (Ka:Ks >1). These results in conjunction with the non-significant SSD model values obtained from mismatch distribution tests suggest that this gene may be under positive selection in both populations. Although the ITS region is transcribed, putatively conserved domains as a translated protein were not detected and no coding regions were identified thus, the Ka:Ks ratio could not be determined. However, the demographic data and the large significant negative values obtained for Fu and Li’s D* statistic for both populations, suggest that the ITS region may also be under selection.

Conservative estimates of the putative time of population expansion from tau (t), calculated by ARLEQUIN after 1,000 re-samplings, indicate that the Trinidad population has a more recent evolutionary history and that the Mexico population is older and would have diverged earlier than the Trinidad population based on analysis of ACT, GPDH and β-TUB gene regions.

The sum of squared deviations (SSD) of mismatch distribution was significant for ACT and β-TUB gene regions of the Mexico *C. truncatum* population indicating a fit to the demographic expansion model tested. The sum of squared deviations (SSD) of mismatch distribution was not significant for the GPDH and ITS gene regions of the Mexico population which provides evidence to reject the null hypothesis of recent population expansion (Table 7(b)).

**Table 7:** **Neutrality tests for Trinidad and Mexico populations of (a) *Colletotrichum gloeosporioides sensu lato *and (b) *C. truncatum***

	**ACT**		**GPDH**		**β-TUB**		**ITS**	
	**Trinidad**	**Mexico**	**Trinidad**	**Mexico**	**Trinidad**	**Mexico**	**Trinidad**	**Mexico**
(a)								
**Tajima’s *D *between populations**	−2.07105*		−2.01052*		−1.04651		−2.17403*	
**Tajima’s *D***	1.94627**	0.45602	1.38271*	1.63479**	2.06785	1.23315	2.0617'***'	−0.28303
Fu and Li’s F* statistic	3.30308**	0.5991	−2.41267	−2.44380*	1.88961**	−0.19344	4.32360**'	−0.07668
Fu and Li’s D* statistic	−3.11602*	0.5439	−2.29389	−2.55070*	1.14719	−0.10131	4.29263**'	1.42066*
**SSD**	0.03231	0.04492	0.0304	0.13253	0.19561**	0.13447***	0.0853***	0.06698***
(b)								
**Tajima’s *D *overall**	0.66745		−0.52899		2.68162**'		0.27089	
**Tajima’s *D***	2.10118	−1.11173	NC	0.01499	NC	1.90106**'	NC	1.03446
Fu and Li’s F* statistic	1.64330*'	−1.34668	NC	0.80424	NC	2.24967**'	NC	0.96179
Fu and Li’s D* statistic	1.31251	−1.24341	NC	0.68403	NC	2.43968**'	NC	−0.8049
**SSD**	0.37279	0.05146*	NC	0.00407	NC	0.35852***	NC	0.03788

* *P* ≤ 0.05.

** *P* ≤ 0.02 for F* and D* statistic.

*** *P* ≤ 0.001.

*NC* Not computed.

### Migration estimates

Historical migration between populations was estimated. For *C. gloeosporioides sensu lato* isolates, there was evidence of asymmetrical migration from Mexico to Trinidad for three gene regions (ACT, GPDH and ITS) as migration rates were higher for the Mexico to Trinidad direction than in the opposite direction. β-TUB was shown to be under positive selection and would not be exchanged in gene flow events. The effective population size was also larger for Mexico than for Trinidad. The number of immigrants was higher for the Trinidad population for the three genes than for the Mexico population. The 2.5%, 97.5% and the mean likelihood estimates are presented (Table 8). Gene flow estimates from *G*_ST_ values corresponded to migration patterns deduced by MIGRATE.

**Table 8:** **Migration patterns for Trinidad and Mexico populations of *Colletotrichum gloeosporioides sensu lato***

			***M *(m/mμ)**	
	**Population**	**xNemμ**^**1**^	**1,+ (Mexico)**	**2,+ (Trinidad)**
**ACT**	Mexico	0.2213 (0.1221 to 0.0.4490)	…	83.120 (68.6375 to 109.01)
	Trinidad	0.1033 (0.0640 to 0.2870)	119.95 (93.4173 to 144.16)	…
**GPDH**	Mexico	0.2189 (0.0872 to 0.5354)	…	98.1445 (63.5554 to 114.73)
	Trinidad	0.1117 (0.0671 to 0.2625)	125.06 (105.44 to 146.80)	…
**β-TUB**	Mexico	0.0079 (0.0028to 0.0179)	…	146.73 (6.7084 to 881.68)
	Trinidad	0.0023 (0.0009 to 0.0054)	3.84 e-010 (1.92e-010 to 113.01)	…
**ITS**	Mexico	0.0126 (0.0069 to 0.0238)	…	56.533 (6.2661 to 180.15)
	Trinidad	0.0140 (0.0067 to 0.0303)	416.6 (142.42 to 1.01e+003)	…

2.5% to 97.5% confidence intervals given in parentheses.

^1^ x scalar = 2 for haploids.

## Discussion

The objectives of this study were to (i) compare the genetic and population structure of isolates of *C. gloeosporioides sensu lato* and *C. truncatum* from Mexico and Trinidad and (ii) determine the migration pattern of these isolates based on multi-locus sequence data.

A higher degree of haplotype and nucleotide diversity is expected in an ancestral population [33-35]. We found greater nucleotide diversity in the Mexico *C. gloeosporioides sensu lato* population for all genes and greater haplotype diversity in the Trinidad *C. gloeosporioides sensu lato* population for ACT and GPDH genes alone. The measures of haplotype diversity, *H*d, can range from zero, meaning no diversity, to 1.000, which indicates high levels of haplotype diversity [34]. With respect to the Mexico population, all four genes had a haplotype diversity index that ranged from 0.768 to 0.863 indicating high levels of diversity for each gene. With respect to the Trinidad population, all four genes had a haplotype diversity index that ranged from 0.576 to 0.922 indicating a wider range of diversity spanning moderate to high levels of diversity for each gene. If Mexico was the older ancestral population and Trinidad the more recent population, then there would be more time for accumulation of nucleotide changes and nucleotide diversity in Mexico compared to Trinidad. This would explain the differences in nucleotide diversity in the two populations. Further, if gene flow occurred and was contemporary and recurrent, then the haplotype characteristics would be similar for both populations. Because there was only one shared haplotype (for the GPDH gene) between the two populations, gene flow is likely to have been a past event. Another explanation for the differences in haplotype characteristics would be that more intensive sampling may be required to detect shared haplotypes, however, this may be unlikely as the same pattern of haplotype dissimilarity was found for all four gene regions. Gene flow is unlikely to have contributed significantly to the observed haplotype distribution across coasts. We interpreted this gene flow between *C. gloeosporioides sensu lato* populations as an historical event that occurred prior to isolation and divergence.

Divergent selection can impose variable genome-wide effects. For example, gene flow can override genetic differentiation at all loci except those that are directly under selection or are linked to selection [36,37]. This explains the higher level of genetic differentiation (based on Nei’s *G*_ST_ values) in the β-TUB gene region which was under selection and was not exchanged in gene flow events.

We also examined the data for the occurrence of sub-division in our study isolates based Bayesian posterior probability, AMOVA, and Hudson’s test statistics. We found significant within-population sub-division of the Trinidad and Mexico *C. gloeosporioides sensu lato* populations and low levels of genetic divergence among populations for three of the four gene regions (β-TUB was the exception). Hudson’s *S*_nn_ statistic indicated that the *C. truncatum* populations were highly sub-divided for ACT, β-TUB and ITS gene regions.

Mutation rates are usually low but can vary according to loci and pathogen and the effects are detectable when operating in conjunction with other evolutionary forces e.g. population size [31]. In this study, Watterson’s estimator of mutation rate indicated that mutation may have some role in shaping the population structure of *C. gloeosporioides sensu lato* isolates in Trinidad and in Mexico especially with respect to the ACT and GPDH gene regions. The lowest number of mutations was obtained for the β-TUB gene region which is expected as this region was shown to be under positive selection. Strong positive selection causes a reduction in levels of nucleotide diversity (37) and the β-TUB gene region had the lowest level of nucleotide diversity of all four gene regions in the Trinidad population. Mutation in the ITS region would be tolerated once the transcribed product is not negatively affected and compensation mechanisms would be engaged because of the multi-copy number nature of this region.

Linkage disequilibrium (LD) can be influenced by a number of evolutionary factors including selection by selective sweeps in which the alleles at flanking a locus under selection are rapidly swept to high frequency or fixation due to genetic hitchhiking [32]. Population expansion can also affect LD as large populations could maintain genetic diversity generated by past recombination events even if they occurred rarely with weakly acting genetic drift to reduce variation. Population expansion has been detected for both populations in this study. Generally, the effective population size was also found to be greater for the Mexico population. If there is LD between loci in the source population, then this will contribute even further to non-random association of alleles which may explain why the Trinidad *C. gloeosporioides sensu lato* population appeared to have a higher LD than the Mexico population.

To further examine evolutionary forces acting on the populations of *C. gloeosporioides sensu lato* three neutrality test statistics (Tajima’s *D*, and Fu and Li’s D* and F*) were used in this study to examine the sequence data for departure from neutrality. D* and F* test statistics for all except the β-TUB gene were significant and negative in the Trinidad population, which we cautiously interpreted as rejection of the null hypothesis of constant population size. Other evolutionary processes such as genetic hitchhiking or extinction and recolonization events [31,32] can cause fluctuation in population size. Additional analyses may be necessary to discriminate between the two competing alternative hypotheses that may explain changes in population size.

The Mexico population of *C. truncatum* isolates had higher haplotype and nucleotide diversity than the Trinidad population. But, again, there was only one shared haplotype (for the GPDH gene region) between the two populations. There was no evidence of gene flow, however, for these populations and it is likely that the haplotype characteristics reflect distinct populations that shared no past or historical migration events. Watterson’s estimator of mutation rate was also lower for all four genes for *C. truncatum* isolates than for *C. gloeosporioides sensu lato* isolates. It is likely that the *C. truncatum* populations emerged independently of each other. Tapia-Tussel et al. [12] suggested that co-cultivation of pepper and papaya may have given rise to isolates with the ability to infect and cause disease in papaya. In Trinidad, co-cultivation of both host species is also common. Further research on tracking the migration of *C. truncatum* infecting a range of different hosts (including pepper) would reveal more information of the possible introduction of this species to Trinidad and Mexico.

Structural and metabolic genes that are transcribed and then translated into protein products are often used in phylogenetic analysis and for taxonomic demarcation, but their use in population genetics studies may result in a bias in the evolution of genes that are under strong selection. Nucleotides in third or wobble codon position may not be subject to positive selection but, their frequency in a given population could be affected by proximity to selected regions through genetic hitchhiking [29,30]. In smaller and younger populations, there is also a question of insufficient variation in the genes to make substantiated explanations with respect to population genetics. The study of a single gene is also of particular concern because its genealogy may not truly reflect the history of populations or species under study and would lead to erroneous conclusions.

The relative migration rates of hosts and pathogens play a key role in determining patterns of local adaptation, which can in turn influence patterns of disease. Migration influences the evolution and sustainability of genetic diversity in host resistance, pathogen virulence genes, and fungicide resistance [29-31]. It is valuable to explore and track the co-evolutionary dynamics of host-pathogen systems. The rational use of integrated management strategies requires an understanding of pathogen adaptive dynamics. Several evolutionary phenomena, such as genetic drift, migration, and selective pressure make it possible for an escape mutant to emerge [29]. Understanding the evolution of epidemics, therefore, can lead to essential changes in the criteria for disease and quarantine control. Such data are needed to predict the emergence, genetic and population structure, and differential pathogen responses to treatment especially in highly diverse and complex ecological landscapes like the agricultural setting.

## Conclusions

With respect to *C. gloeosporioides sensu lato* isolates, population expansion has been detected for both populations in this study, however, the effective population size was found to be greater for the Mexico population. There was also evidence of asymmetrical migration from Mexico to Trinidad for three gene regions (ACT, GPDH and ITS). Mexico appeared to be the older ancestral population and Trinidad the more recent population based on the estimated demographic parameters. This would explain the differences in nucleotide diversity in the two populations. There were also dissimilar haplotype characteristics for both populations. Watterson’s estimator of mutation rate indicated that mutation may have some role in shaping the population structure of *C. gloeosporioides sensu lato* isolates in Trinidad and in Mexico especially with respect to the ACT and GPDH gene regions. The data suggests that *C. gloeosporioides sensu lato* isolates may have co-migrated with the host to Trinidad. There was no evidence of gene flow between the *C. truncatum* populations and it is possible that these populations emerged independently of each other.

Pathogen populations may be bound by differently-acting evolutionary processes including recent or historical migration events, selective sweeps and extinction-recolonization processes typical of agroecosystems [31]. Strong priority effects may enable genetic differentiation that is sufficient to move populations along independent evolutionary routes. Understanding the genetic structure of pathogen populations will assist in determining the evolutionary potential of the pathogen and in identifying which evolutionary forces may have the greatest impact on durability of resistance. Intervention strategies that target these evolutionary forces would prove to be the most practical.

## Methods

### Collection and maintenance of isolates

Papaya fields in the main production areas in Trinidad and Mexico were surveyed at their harvesting stage during the period November 2010 to April 2011. Symptomatic fruit were placed in separate bags and transported to the laboratory. Fruit were surface sterilized by rinsing with 70% ethanol for 2 min, followed by three rinses with sterile distilled water. Samples were then blotted dry and placed onto individual sterile petri-dishes. A 4 mm^3^ block of tissue was removed from advancing edge of each lesion and was placed in the centre of 2% water agar -WA (BactoAgar, Difco Ltd., USA) amended with 50 mg L^-1^ streptomycin and 50 mg L^-1^ tetracycline (Sigma-Aldrich Co. Ltd. USA). Plates were incubated for five days at 25°C in the dark. After incubation, a block of agar (4 mm^3^) taken from the advancing mycelial edge of an actively growing culture was removed and placed in the centre of a PDA plate (Potato dextrose agar -PDA, Oxoid Ltd., UK) supplemented with 50 mg L^-1^ streptomycin and 50 mg L^-1^ tetracycline). Cultures were incubated for five days at 25°C in the dark. Isolates were maintained as conidial suspensions in 50% glycerol at −70°C for long-term storage (Table 1 Summary of isolate collection data for Trinidad and Mexico”).

### DNA extraction, PCR, and sequencing

Isolates were grown in PDB (potato dextrose broth) in the dark for nine days at 25°C in a rotary shaker at 150 rpm. The mycelial mat was removed and dried on sterile filter paper. DNA was extracted using the E.Z.N.A. DNA extraction kit (Omega bio-tek Ltd., USA). Four gene regions were amplified by PCR: ITS rDNA, partial β-tubulin (β-TUB), partial actin (ACT) and glycerol-3-phosphate dehydrogenase (GPDH) gene regions. PCR reactions were carried out using the universal primer pair ITS4/5 to amplify the ITS region (496 bp) of the nuclear ITS1-5.8S-ITS2 rDNA [38], Bt2a/b primers for amplifcation of a 560 bp fragment of the β-tubulin gene [39], and GPDH and ACT specific primers [40,41] to amplify 300 bp and 290 bp fragments respectively. For each 25 μL reaction, PCR components (Invitrogen by Life Technologies Co., USA) included 1 × PCR buffer; 1.5 mM MgCl_2_, 0.2 mM dNTP, 2.5 U Taq DNA Polymerase and 50 pmoles of each primer (Integrated DNA Technologies, USA). PCR amplification conditions consisted of an initial denaturation of 5 min at 94°C followed by 35 cycles of 1 min at 94°C, 1 min at 55°C, 1 min at 72°C with a final extension of 5 min at 72°C. PCR products were sequenced directly (Amplicon Express, WA, USA and Macrogen Ltd., Korea).

The identity of the sequences of isolates and comparisons of cognate sequences available in the GenBank and EMBL public databases were made using the gapped BLASTn algorithm. Sequences of all isolates were compared to cognate sequences of epitype and ex-epitype strains of *C. gloeosporioides* and were identified as belonging to the *C. gloeosporioides sensu lato* circumscription before inclusion in subsequent analyses. Sequences of representative isolates were deposited in GenBank. A total of 204 sequences was used in the final dataset for *C. gloeosporioides sensu lato* isolates: 120 sequences from the Trinidad population (*n* = 30 for each of the four gene regions) and 68 sequences from the Mexico population (*n* = 17 for each of the four gene regions). A total of 84 sequences was used in the final dataset for *C. truncatum* isolates: 32 sequences from the Trinidad population (*n* = 8 for each of the four gene regions) and 52 sequences from the Mexico population (*n* = 13 for each of the four gene regions).

### Multilocus sequence analysis

Alignments were performed using MUSCLE [42] under default parameters. Using the Se-AL programme (http://tree.bio.ed.ac.uk), the alignment was then visually inspected, edited, and trimmed to common nucleotide lengths for ACT, GPDH, β-TUB and ITS gene regions.

### DNA divergence

Standard population genetic analyses were performed using DNASP version 5.10 [43,44] and ARLEQUIN version 3.1 [45]. There were no *a priori* assumptions about the isolates and their assignments to populations or sub-populations other than in the operational sense as isolates collected from a particular country i.e. isolates collected from Mexico constituted the Mexico population and those collected from Trinidad constituted the Trinidad population.

Measures of DNA divergence including the average number of nucleotide differences (*k*), nucleotide diversity (pi and pi(JC)) for each population were computed. Watterson’s estimator of mutation rate (*θ*-W) on a per site and per sequence basis was also calculated for all four genes for each population. The conservation threshold was also determined.

Haplotype diversity was estimated for each gene and population, however, genealogies from haplotypic genetic data based on statistical parsimony approaches were not estimated because of there were no shared haplotypes for three of the four genes [46].

### Genetic differentiation and population structure

Genetic differentiation between pairs of populations (*G*_ST_) [34] adapted for DNA sequence data [43,44] was calculated and used as a preliminary estimate of gene flow using DNASP. A hierarchical analysis of molecular variance (AMOVA) was carried out to partition total variance into variance components attributable to within and between population differences and significance was assessed using 9,999 permutations of the original data using GenAlEx version 6.3 [47]. Population differentiation as a result of isolation-by-distance model was estimated based on Mantel’s test using GenAlEx assuming a linear relationship between genetic and geographic distances between populations. The significance of this relationship was estimated with 9,999 permutations.

The nearest-neighbor statistic (*S*_nn_) is a measure of how often the “nearest neighbors” (in sequence space) of sequences are from the same locality in geographic space. The statistic is applicable when genetic data are collected on individuals sampled from two or more localities. If a population is strongly structured, one expects to find the nearest neighbor of a sequence in the same locality. Thus, *S*_nn_ is expected to be near 1.0 when the populations at the two localities are highly structured because the majority of sequences would be similar to other sequences of the same population and near 0.5 when the populations at the two localities are not genetically structured because the most similar sequences could come from either population with equal probability [48,49].

*In silico* PCR-RFLP for population sub-division analysis was carried out. Computer-simulated RFLP analysis of the sequences of all amplified gene regions was carried out using NEBCutter V.2 [50] and WEB-Cutter V.2 (http://bio.lundberg.gu.se/cutter2) according to the method of Ramdeen and Rampersad [51]. Twelve restriction enzymes were screened: ApaI, BamHI, DpnI, HaeIII, HindIII, HinfI, MseI, PstI, PvuII, RsaI, SmaI, and TaqI. After *in silico* restriction digestion, a virtual 1.5% agarose gel electrophoresis image was captured as a device-independent plot by the program pDRAW32 (http://www.acaclone.com, [52]). Binary matrices were prepared and an assignment test based on Bayesian posterior probability implemented in STRUCTURE version 2.3.2.1 [53-55] was used to determine sub-population structuring. Ten runs with a burn-in period of 50,000 generations and 100,000 MCMC (Markov chain Monte Carlo) iterations from *K* = 1 to *K* = 5; the model does not identify *K* = 1. This program calculates the membership coefficients to each of the populations (*Q)* of every sample [55].

### Linkage disequilibrium

Linkage disequilibrium (LD) [56] was determined as the number of significant pairwise comparisons based on Fishers’ exact test (*P* ≤ 0.05). The test was implemented in DNASP. Two loci were considered to be in LD when their associated P-value was less than 0.05. Only parsimony informative polymorphic sites were considered.

### Population size changes

To determine whether a model of the population expansion was applicable to both populations we performed the neutrality tests of Tajima’s *D*[51] and Fu and Li’s D* and F*[57-59] statistics to estimate deviations from selective neutrality. Zeng et al. [60] pointed out that there are important aspects of the data that Tajima’s *D* does not consider. As a result, it may be less powerful and may not be as efficient at detecting departures from neutrality as other alternative statistics hence the reason for implementing Fu and Li’s tests. Neutrality tests were used as an indication of recent population expansion when the null hypothesis of neutrality was rejected due to significant negative values where Tajima’s *D* statistic was significant at *P* ≤ 0.05; and Fu and Li’s D* and F* statistic were significant at *P* ≤ 0.02 [58] under 10,000 coalescent simulations with the software DNASP. The significance of Tajima’s *D* was tested by random permutation using 1,000 replicates in ARLEQUIN. Significantly negative values of these neutrality statistics although indicative of population expansion, may be due to other evolutionary forces such as background selection and genetic hitch-hiking associated with selective sweeps.

We also used analysis of mismatch distribution [61,62] to help visualize signatures of demographic expansion for all the samples combined, and to test the null hypothesis of population growth. To test whether the observed distributions deviated significantly from those expected under the population expansion model, we computed the significance of sum of squared deviations (SSD) (with 1,000 replications) for each gene and for each population using ARLEQUIN.

We estimated the putative time of population expansion most of the populations from the tau (t) statistic (expressed in units of mutational time) which was calculated in ARLEQUIN. An estimate of Tau = 2ut is given where u = mTμ and t is the estimated time of expansion, mT is the number of nucleotide sequences under study and μ is the mutation rate per time.

The divergence time (tau) between populations of unequal size was estimated [63]. The model assumes that two populations have diverged from an ancestral population of size N_0_ some T generations in the past and have remained isolated from each other ever since. From the average number of pairwise difference between and within populations the divergence time scaled by mutation rate is estimated and a conservative interpretation can be explained [45].

### Test s for migration

Historical migration between populations was estimated with MIGRATE-n version 3.0.3 [64]. MIGRATE is based on coalescent theory to estimate effective population sizes (theta = θ, also called population diversity) and allows for estimation of asymmetrical migration (*M*) between population pairs. Estimates of gene flow among populations were obtained using the maximum likelihood approach, full migration matrix model of 10 short chains, each with a total of 50,000 genealogies and with a sampling increment of 100 genealogies and 10 long chains, each with a total of 100,000 genealogies and a sampling increment of 100 genealogies. The first 10,000 genealogies in each chain were discarded. All other settings were default to the program. The confidence interval for *θ* and migration parameter *M* was calculated using a percentile approach [64]. The program assumes on-going exchange of migrants, therefore, if populations are isolated, then the calculated migration rate and direction would be a reflection of the populations prior to separation.

## Availability of supporting data

The data set supporting the results of this article is available in the LabArchives LLC repository, http://dx.doi.org/10.6070/H4BZ63Z8.

Representative sequences, as their assigned GenBank accession numbers, can be accessed using the following links:-

JF749805, http://www.ncbi.nlm.nih.gov/nuccore/JF749805

JF749806, http://www.ncbi.nlm.nih.gov/nuccore/JF749806

JQ218143, http://www.ncbi.nlm.nih.gov/nuccore/JQ218143

JQ218144, http://www.ncbi.nlm.nih.gov/nuccore/JQ218144

JQ218145, http://www.ncbi.nlm.nih.gov/nuccore/JQ218145

JQ218146, http://www.ncbi.nlm.nih.gov/nuccore/JQ218146

HQ287579, http://www.ncbi.nlm.nih.gov/nuccore/HQ287579

HQ287580, http://www.ncbi.nlm.nih.gov/nuccore/HQ287580

HQ287581, http://www.ncbi.nlm.nih.gov/nuccore/HQ287581

HM450128, http://www.ncbi.nlm.nih.gov/nuccore/HM450128

HM450129, http://www.ncbi.nlm.nih.gov/nuccore/HM450129

HM562709, http://www.ncbi.nlm.nih.gov/nuccore/HM562709

HM562710, http://www.ncbi.nlm.nih.gov/nuccore/HM562710

HM562711, http://www.ncbi.nlm.nih.gov/nuccore/HM562711

JX982230, http://www.ncbi.nlm.nih.gov/nuccore/JX982230

JX870645, http://www.ncbi.nlm.nih.gov/nuccore/JX870645

JX878500, http://www.ncbi.nlm.nih.gov/nuccore/JX878500

JX870646, http://www.ncbi.nlm.nih.gov/nuccore/JX870646

## Competing interests

The authors declare that they have no competing interests.

## Authors’ contributions

SR collected the fungal isolates and performed the experiments for Trinidad; DP-B, CT-C and RT-T collected the fungal isolates and performed the experiments for Mexico. CC carried out all of the alignments. SR analyzed the data and wrote the manuscript. All authors read and approved the final manuscript.
